# Quantitating the subtleties of microglial morphology with fractal analysis

**DOI:** 10.3389/fncel.2013.00003

**Published:** 2013-01-30

**Authors:** Audrey Karperien, Helmut Ahammer, Herbert F. Jelinek

**Affiliations:** ^1^Centre for Research in Complex Systems, School of Community Health, Charles Sturt UniversityAlbury, NSW, Australia; ^2^Institute of Biophysics, Medical University of GrazGraz, Austria; ^3^Rural Clinical School, University of New South WalesSydney, NSW, Australia

**Keywords:** microglia, cell shape, image interpretation: computer-assisted, fractals, models: biological, box counting, lacunarity, multifractal analysis

## Abstract

It is well established that microglial form and function are inextricably linked. In recent years, the traditional view that microglial form ranges between “ramified resting” and “activated amoeboid” has been emphasized through advancing imaging techniques that point to microglial form being highly dynamic even within the currently accepted morphological categories. Moreover, microglia adopt meaningful intermediate forms between categories, with considerable crossover in function and varying morphologies as they cycle, migrate, wave, phagocytose, and extend and retract fine and gross processes. From a quantitative perspective, it is problematic to measure such variability using traditional methods, but one way of quantitating such detail is through fractal analysis. The techniques of fractal analysis have been used for quantitating microglial morphology, to categorize gross differences but also to differentiate subtle differences (e.g., amongst ramified cells). Multifractal analysis in particular is one technique of fractal analysis that may be useful for identifying intermediate forms. Here we review current trends and methods of fractal analysis, focusing on box counting analysis, including lacunarity and multifractal analysis, as applied to microglial morphology.

## The form-function connection

Microglia, small in size but enormous in significance, occupy a conspicuous space in presumably all nervous systems. They interweave intimately and abundantly with the generally much larger neurons at a ratio that can be said to average one to one in normal adult human tissue, but depends on when and from where in the central nervous system (CNS) the tissue sample is taken. The 3-dimensional space microglia occupy constantly changes as they move their cell processes and migrate, but if caught in stop motion, microglia can be seen to come in an extraordinary variety of intricate and complex morphologies (Dowding et al., 1991; Dowding and Scholes, 1993; Sonetti et al., 1994; Magazine et al., 1996; Dobrenis, 1998; Perry, 1998; Alliot et al., 1999; Bernhardi and Nicholls, 1999; McMenamin, 1999; Streit et al., 1999; Navascues et al., 2000).

**Figure 1:**
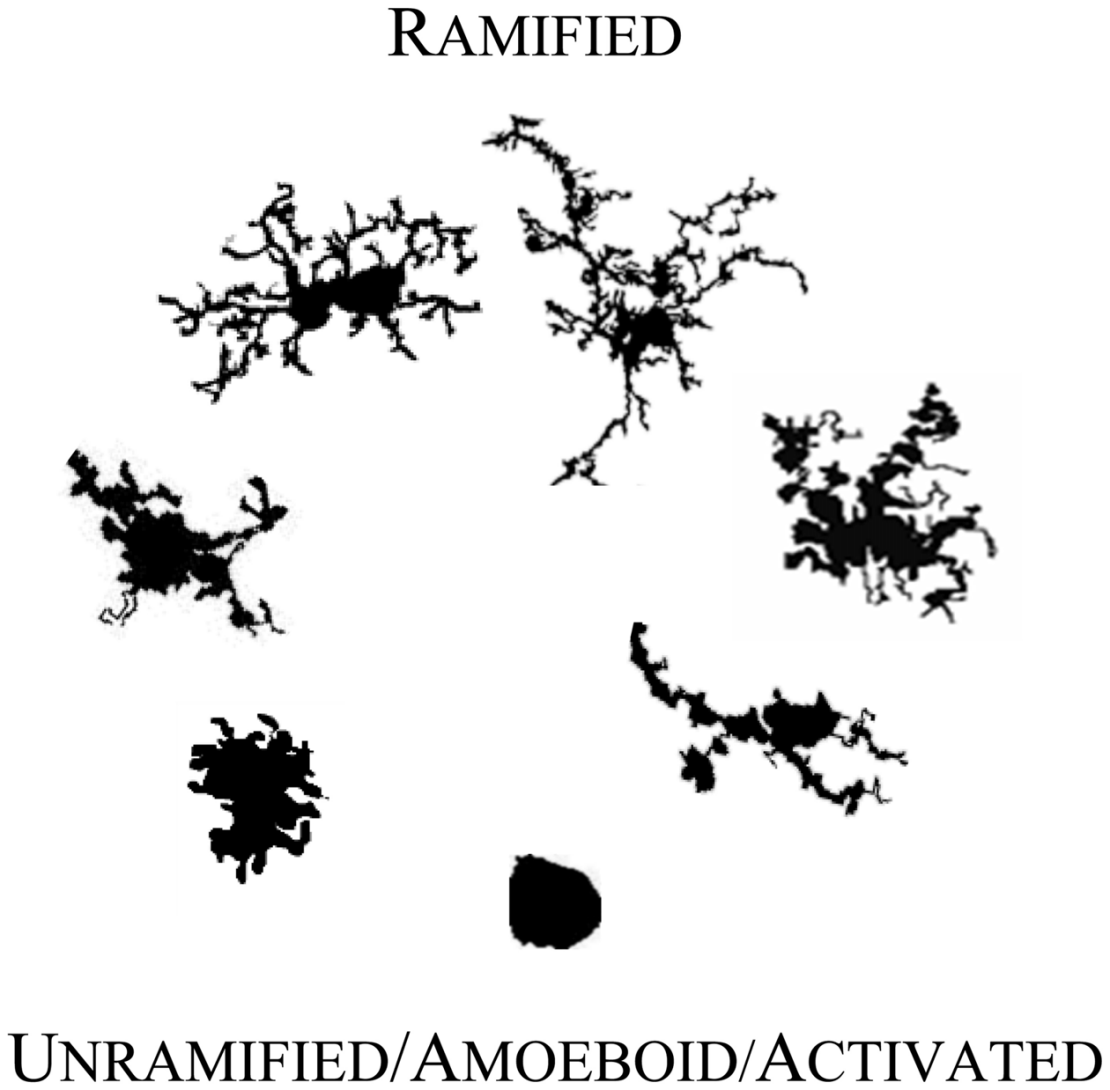
**Microglial morphology in adult human CNS**. Microglia are morphologically and functionally dynamic cells able to change form from highly ramified to completely lacking processes. The transition can be very rapid or microglia can remain in a form for years (Colton et al., 2000). The forms illustrated here represent snapshots of a transformation that is reversible at every point, with variation within each form shown. The figures are not shown to scale; they are adjusted to compare detail.

As Figure 1 illustrates, individual microglial cells can cycle reversibly from a simple rounded to a complex branched form. At any point in time they might be found as round to amorphous blobs with any of a variety of intriguing membrane features such as pseudopodia and ruffles. Or at the other extreme, they may adopt a form with a relatively very small soma and long, tortuous primary processes that can be characterized as “spider-like,” “jointed,” or “thorny” with secondary and tertiary branches endowed with wispy ends or yet further branches (Kreutzberg, 1995; Dailey and Waite, 1999; Ohsawa et al., 2000; Streit, 2000; Bohatschek et al., 2001; Nimmerjahn et al., 2005; Tremblay et al., 2011; Liu et al., 2012).

On the basis of this highly flexible morphology, neuropathologists have fashioned a model linking names for microglial forms to microglial function. In the mature CNS, microglia in their unramified and intermediate forms (moving upward from the bottom of Figure 1) are generally considered to be “activated,” “reactive,” or “intermediate.” They are activated for an immunoinflammatory role that includes traveling to sites of injury where they can recruit or activate other cells, proliferate, phagocytose, clear debris, and contribute to healing and cortical reorganization. They may also express a typical immunoinflammatory profile such as upregulated CD68 and major histocompatibility complex II proteins (MHC-II) (Streit and Kreutzberg, 1987). Seen in a dish, on a slide, or in a live organism, microglia thus may appear plump, dragging and heaving corpulent bodies, or perhaps scalloping at the edges. They may be rapidly protruding in and out a few or many stout processes, and may simultaneously be, a little more slowly, winding in their finer arbor. In this role, microglial cell bodies can be elongated lumpy, rod-like or tortuous with swollen branching projections, or microglia can be more radial, spiky figures (Streit, 2000; Soltys et al., 2001; Stence et al., 2001).

In their fully ramified forms (atop Figure 1) in normal mature CNS, microglia are actively engaged in essential physiological roles. They are vigilant sentinels and “synaptic partners,” watching over and ensuring proper functioning of neurons, providing neurotrophic substances (Nakajima and Shinichi, 2002), acting on and regulating neurotransmitters and hormones (Garcia-Segura et al., 1996), mediating pain (Watkins et al., 2001; Inoue, 2006) and responses to psychological stress (Hinwood et al., 2012), protecting neurons from damage (Vinet et al., 2012), and responding to changes in the microenvironment (e.g., stretch, depolarization, glycemic status, etc.) (Eder et al., 1998; Lyons and Kettenmann, 1998; Polito et al., 2011; Tremblay et al., 2011; Won et al., 2012).

As they do their multifarious duties, ramified cells change and move in many ways over multiple time scales. Their arbor itself changes as they wriggle and wave, extend and retract fine and gross processes, tend to synapses, migrate, and phagocytose (Pow et al., 1989; Dailey and Waite, 1999; Lee et al., 2008; Marker et al., 2010; Tremblay et al., 2011). Microglia have been observed moving their processes “exuberantly,” more at the ends than near the soma, in seemingly random directions but within a volume so that they maintain a consistent basic symmetry and overall arbor size. They might start to move more rapidly and become more polarized as they modify and extend their processes toward a site of injury, and may migrate toward the site while still ramified. Indeed, they can continue to mark their post and send off a replica if they feel a need to tend to something at a distance (Radewicz et al., 2000; Aarum et al., 2003; Nimmerjahn et al., 2005; Lee et al., 2008; Wake et al., 2009; Perego et al., 2011).

Knowledge of this model is a powerful tool in the neuroscientist's toolkit. It suggests that based on visual impressions of local microglial morphology, much can be inferred about what is going on in a particular location. Such visual impressions do in fact inform the decisions of pathologists and researchers (Streit, 2000). The model is especially powerful because microglia play an integral role in the developing and mature CNS, during pathological and normal states, affecting structure, plasticity, and function in virtually all circumstances. Microglia have been found to mediate effects of and respond to a host of substances as diverse as minocycline (Hinwood et al., 2012), ethanol (Crews et al., 2006; Kane et al., 2011; Zhao et al., 2012), nonsteroidal anti-inflammatories (Varvel et al., 2009), opioids (Wen et al., 2011), cannabinoids (Toth et al., 2010), and neuroleptics (Yrjanheikki et al., 1998; Busse et al., 2012), and are increasingly being seen as potential targets for therapeutic intervention and monitoring of events in the nervous system (Billiards et al., 2006; Liu et al., 2012; Pascual et al., 2012).

However powerful this form-function model is, it is also limited. Applying it too generally can gloss over important features of intermediate forms between categories, subtle variation within a category, and the considerable crossover in function amongst cells in different categories. Ramified cells were historically characterized as “resting,” for example, from observations of fixed microscope slides, but advancing imaging techniques including thinned-skull, live imaging revealed that ramified microglia are “resting” only from their alternative starring role in emergency response. Indeed, the moniker “never resting microglia” is rising in popularity amongst microgliologists. Microglia were also thought of as unable to respond to compromise without being morphologically activated, but this was another of our assumptions advancing research revealed to be wrong. In sum, it has proven extremely useful in many ways but in others inadequate to infer function from biochemical markers and preconceived morphological categories. So, the model must continue to grow. The investigator who is attempting to unravel brain function, track responses to treatments, and detect pathological changes must remember that the form-function model is a starting point, and be vigilant to the possibility of multifarious factors affecting microglial morphology and function (Radewicz et al., 2000; Aarum et al., 2003; Nimmerjahn et al., 2005; Wake et al., 2009; Perego et al., 2011).

In doing so, the microgliologist might also note that the form-function model is poor at quantitating subtle morphological differences. At what point should one consider a cell to have crossed over from ramified to activated, for instance? In a still photo, how should one decide if a cell is de- or re-ramifying? How should an investigator decide if the subtle difference in branching angle between two cells is part of the puzzle they are trying to solve? Or how should one classify a cell with a very large soma but a single, long, slender, branching process, and what should one assume such a cell is doing based on traditional classification systems? On another level, how should one interpret an area with half of the microglia in one category and the other half in another? These questions are more than academic when it comes to both practice and research. Microglia undergo changes in a host of domains along multiple time scales (e.g., soma size and shape, relative cytoplasmic volume, membrane configuration, receptor distribution, cytoskeletal organization, process length, diameter, and degree of branching, branch configuration and tortuosity). These are challenging to distil into practical metrics using traditional objective measuring methods, but the model is at a point where it needs to become more quantitative.

One method that may contribute to this need is fractal analysis. Thus, the rest of this review comments on how fractal analysis is helping make models of microglial form and function more quantitative. To help the reader interpret fractal analysis studies of microglia, we provide a brief methodological overview. We also discuss how fractal analysis has been used to quantitate microglial morphology and how it might generate hypotheses about the form-function connection in the future. We focus on one particular type of analysis, *box counting*, which has been used to quantify not only gross morphological differences but also subtle nuances of microglial morphology that may be important for understanding normal and pathological CNS.

## Fractal analysis

Fractal analysis is a group of methods for quantifying difficult to describe patterns (Jelinek and Fernandez, 1998). We summarize here only a few of its elements, those necessary to understand the results discussed in this review. Box counting and the box counting dimension are our exemplar for three reasons. First, box counting is exquisitely sensitive to morphological features that are analogous to key structural features of microglia (i.e., branching patterns and contours, respectively analogous to ramified processes and membrane detail) (Losa et al., 1997). Second, it has proven successful for analyzing microglia. And third, box counting software has become increasingly accessible to the neuroscience community (Karperien, 2001a; Baksi and Fidler, 2012). The reader should be aware that alternative fractal methods such as the *dilation method* and *mass radius method* have been used to characterize microglial morphology (Soltys et al., 2001; Orlowski et al., 2003; Varvel et al., 2009), and that others may prove useful (e.g., local connected fractal dimension) (Karperien, 2001a; Losa et al., 2005; Karperien et al., 2008b) but have not yet been tried.

The reader should also be aware that a dearth of data exists on fractal analysis of microglia in general. As computerized methods of image analysis have burgeoned in the last decades, fractal analysis in neuroscience has grown to include many applications ranging from classifying neural cells to assessing diabetic retinopathy (Smith et al., 1996; Fernandez and Jelinek, 2001; Karperien et al., 2008b; Jelinek et al., 2010; Kim et al., 2011). As substantial as this body of literature is, it contains only a very small number of studies reporting on fractal analysis of microglia, many published by our lab using *FracLac for ImageJ*. FracLac is open-source software freely available to the bioscience community through the ImageJ website at the National Institutes of Health. It was developed by our lab to control and automate fractal analysis of microglia and provide complementary measures of cell morphology (Karperien, 2001a; Mancardi et al., 2008; Kam et al., 2009; Sant and Fairgrieve, 2012; Schneider et al., 2012).

### Fractal dimensions

At the heart of fractal analysis is the concept of a *fractal dimension* (D_F_). A D_F_ is a number describing how the detail in a pattern changes as the pattern is examined at varying scales. This scaling is generally referred to as *complexity*. The higher the dimension, the more complex the pattern. This is not to say that one would expect to be able to characterize microglia along an infinite spectrum; rather, from a practical perspective, one can generally expect that for 2-dimensional patterns, calculated D_F_s will generally fall between 1 and 2, and for 3-dimensional, between 2 and 3.

Finding a D_F_ for a structure is similar to zooming in with a microscope to examine tissue at different magnifications, but with an important difference. Normally, as one zooms in, one sees at a finer resolution the more fundamental building blocks of a structure; but for a fractal pattern, with each increase in magnification the observer finds the original structure composed of parts identical to itself, just smaller. This *self-similarity* stands out because it is detailed, as opposed to the uninteresting self-similarity in the curve of a simple circle, for instance, which has a theoretical D_F_ of 1.0 and would forever be seen to be made up of merely smaller curves. Moreover, the number of new parts within a structure changes consistently with the scale, such that there is a predictable ratio of new parts to scale, which is the mathematical basis of the D_F_. As shown in Equations 1 and 2, the D_F_ is the exponent to which scale (ε) is raised to get the number of new parts (*N*_ε_):

(1) $$N_{\varepsilon }=\varepsilon^{{\text{D}}_{\text{F}}}$$

(2) $${\text{D}}_{\text{F}}=\frac{\ln N_{\varepsilon }}{\ln\varepsilon }$$

**Figure 2:**
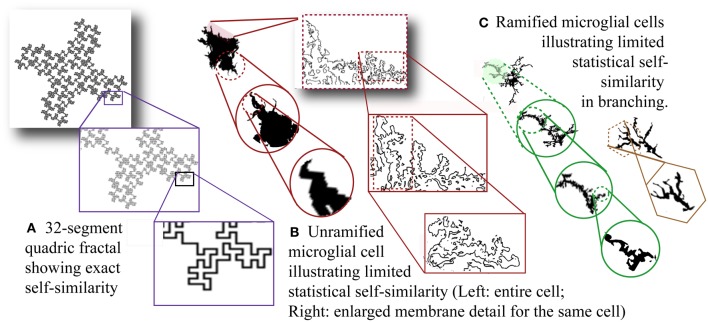
**Self-similarity and scale invariance. (A)** Thirty-two-segment quadric fractal contour illustrating exact but limited self-similarity. The pattern always resolves into 32 new parts each 1/8 of the previous size so the fractal dimension is ln 32/ln 8 = 1.67. The theoretical pattern is infinitely self-similar, but the image is limited by the smallest possible line size that can be used to draw it. [Image generated with ImageJ (Schneider et al.), fractal generator plugin. http://imagej.nih.gov/ij/plugins/fractal-generator.html. Modified from http://rsb.info.nih.gov/ij/plugins/fraclac/FLHelp/Fractals.htm#32seg.] Silhouettes of **(B)** unramified and **(C)** ramified microglia. [Note that the rightmost figure in (**C)** could be classified as intermediate.] **(B)** Showing membrane detail, and **(C)** showing branching detail, depict scaling that is not exactly self-similar and referred to as scale invariant. Moreover, scaling is only detectable within physical limits and the limits of the methods and media used to reveal morphology including staining and recording methods as well as magnification and resolution (both optical and digital).

Figure 2A illustrates self-similarity in one example of a fractal pattern known as a quadric fractal curve. A practical approximation (*D*) of a D_F_ can be estimated from a sample of information based on the limit as scale decreases:

(3) $$D=\underset{\varepsilon \to 0}{\lim}\left[\frac{\ln N_{\varepsilon }}{\ln\varepsilon }\right]$$

*D* from Equation 3 is usually calculated as the slope over a straight interval of the linear regression line for a sample dataset of *N*_ε_ vs. ε.

Relevant to fractal analysis of microglia and other biological phenomena, both theoretical fractals and biological fractal-like patterns (Figures 2B and C) may have statistical rather than strict self-similarity. Furthermore, although in theory a D_F_ describes an infinite scaling ratio, as Figure 2 shows, physically manifested fractal patterns are limited by physical bounds so a *D* measured for such a pattern is taken to be an approximation of scaling within such bounds (Mandelbrot, 1983).

### Box counting fractal analysis

The prohibitively tedious task of gathering a sample dataset implied by Figure 2 is usually approximated by computer software. The inputs to the software are usually 2-dimensional binary patterns (i.e., black and white images), in which pixels can have one of two values, foreground or background; but 3-dimensional data and grayscale images can also be analyzed (Sheets et al., 2010; Ahammer, 2011; Kim et al., 2011).

In order to gather a dataset, box counting software, in essence, lays successively smaller calibre grids over an image, counting the number of boxes containing any foreground pixels to get a proxy for *N* at each calibre, ε = box size. It may also gather the number of pixels in each box, or in the case of grayscale images, the difference in pixel intensity in each box. After gathering the data, the software calculates the box counting dimension (D_B_) according to Equation 4, accounting for box size as related to scale, then using again a regression line.

(4) $$D=\underset{\varepsilon \to 0}{\lim}\left[\frac{\ln N_{\varepsilon }}{\ln\varepsilon^{-1}}\right]$$

Thus, one can think of a D_B_ found for microglia as the mean rate of change in detail with change in resolution sampled from an image. For more about box counting algorithms and implementations, the reader can see Mandelbrot (1983), Karperien (2001b), or Ristanović et al. (2009).

### Fractal literacy

**Figure 3:**
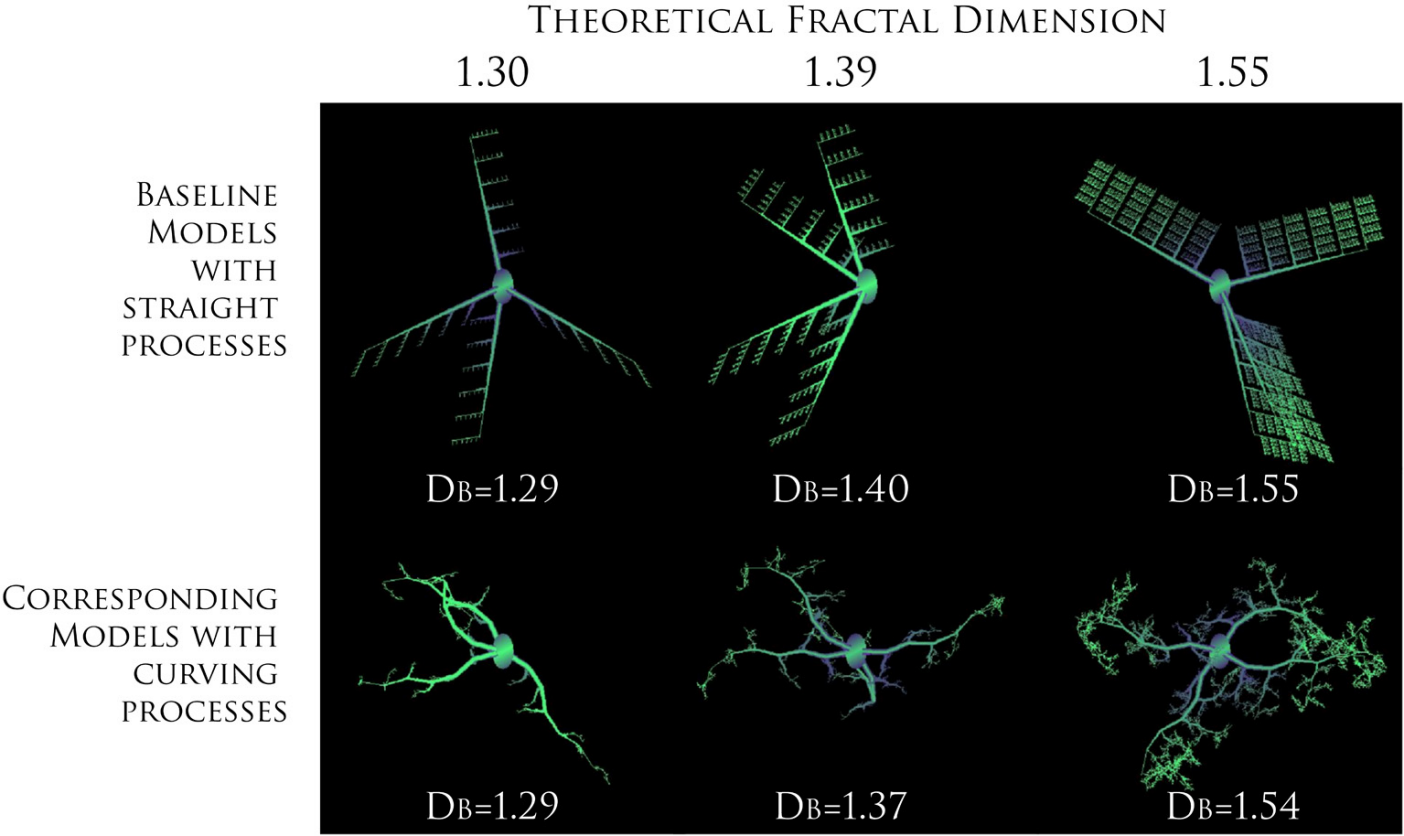
**Increasing complexity of branching patterns**. Branching structures generated by computer based modeling, according to known rules. At each iteration, the ratio of branch length to parent length was changed to illustrate how branching features influence theoretical D_Fs_ and calculated D_B_s. For both series, the direction a branch was allowed to grow in was random. The only difference between the two series was in the amount of curving allowed in processes, which was greater in the images on the bottom. The values for the D_B_ are averages for sets of models rather than only the particular model shown for each series. Modeled with MicroMod, free, open-source modeling software (Karperien, 2001c; Jelinek et al., 2002).

Before going on, we should address two fundamental “fractal literacy” (Jelinek et al., 2005) issues that can have bearing on how one interprets fractal analysis of microglia. First, a D_F_ is neither a unique nor a complete descriptor. To elaborate, although one can generate a set of rules to construct a series of related forms of increasing known complexity that looks like what it is and has calculated D_B_s in agreement with the rules that were used to construct the series (e.g., see Figure 3), one cannot do this in reverse. Knowing a pattern's D_F_ or D_B_ does not tell what the underlying structure looks like, how it was constructed, or how it functions. As a consequence, a box counting algorithm may quantitatively and correctly assess two objects as similar, but the eye may see something very different. As another consequence, a fractal dimension alone cannot describe any feature of microglia. Rather, it is a statistical index of complexity only, a unitless dimension that is entirely independent of traditional measures such as length, area, etc.

Second, box counting neither finds nor confirms the existence of a fundamentally repeating unit; rather, it measures scaling in an image as the averaged dependency between pixel arrangement and box size. The self-similarity in Figure 2 shown for microglia, for instance, was not detected by fractal analysis but was selected for illustrative purposes. An important implication of this point is that being able to determine a D_B_ for a pattern does not mean the phenomenon from which it was gleaned is fractal; neither does a phenomenon have to be fractal to be investigated with box counting fractal analysis (Jelinek et al., 2005).

### Benchmarking

One further point to mention when interpreting fractal analysis studies is that all methods of fractal analysis have limitations. Technical issues specific to box counting, such as effects of grid orientation and calibre, and smoothing the data to find an optimal scaling interval, must be addressed and are generally accounted for within software (Karperien, 2001a; Kam et al., 2009; Sant and Fairgrieve, 2012). In this regard, box counting software is generally validated for any particular analysis using benchmarks having known D_B_s and that are relevant to the images being analyzed (e.g., for microglia, a benchmark would have size, resolution, density of foreground pixels, and pattern features similar to the images being analyzed) (Mandelbrot, 1983; Vicsek, 1992; Karperien, 2001b). Some benchmarks that have been proposed include diffusion limited aggregation and various fractal contours (Karperien, 2004; Jelinek et al., 2011, 2013).

### Supplementary measures: lacunarity and multifractality

**Figure 4:**
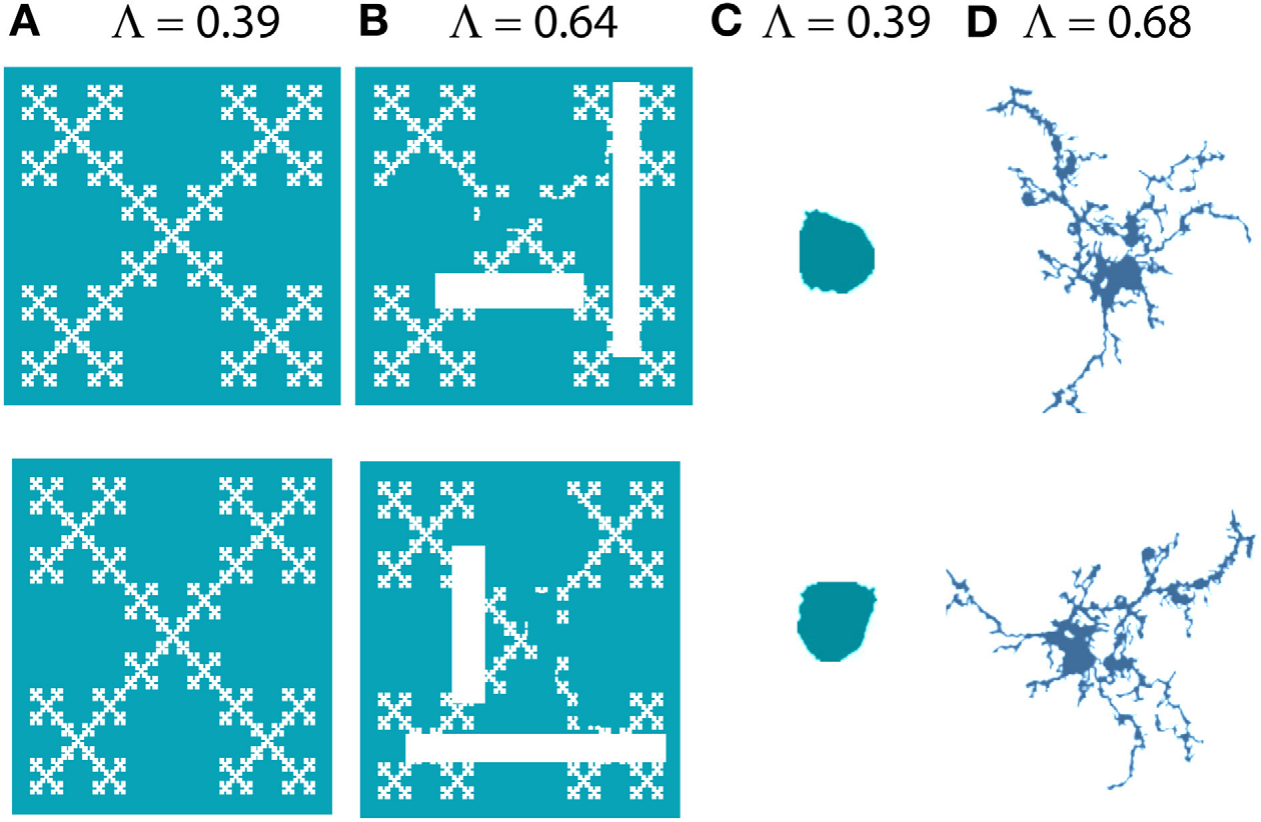
**Lacunarity**. The images on the bottom row are 90° rotations of the images on the top row. Images **(A)** and **(C)** look similar to their originals when rotated, but **(B)** and **(D)** are more affected by rotation; they are more heterogeneous or less rotationally invariant and this is captured and quantified by Λ [e.g., **(B)** has greater lacunarity than **(A)**]. **(A)** and **(B)** were generated with ImageJ fractal generator plugin http://imagej.nih.gov/ij/plugins/fractal-generator.html; **(C)** and **(D)** are from Figure 1.

In addition to fractal dimensions, two other measures obtained from box counting that have been applied to microglia that we will discuss here are lacunarity and multifractality. Both measure scaling of the “mass” or number of pixels per box rather than scaling of the presence of pixels in a box, so if box counting records the number of pixels in each box, these supplementary measures can be calculated as part of the analysis and provide additional features for classification. Whereas the D_B_ measures self-similarity, lacunarity measures nearly the opposite, heterogeneity—patterns with high lacunarity are inhomogeneous and with low lacunarity are homogeneous or rotationally invariant (see Figure 4). There are different ways to define and determine lacunarity; methods that depend on the same data used to calculate a D_F_ are correlated with it and therefore redundant, but those that do not depend on the same data are complementary (Smith et al., 1996). Lacunarity calculated from box counting (Λ), found as the coefficient of variation in pixel density with scale, is independent of the D_B_, and patterns indistinguishable by their D_B_ are often distinguishable by Λ or vice versa (Karperien, 2004; Jelinek et al., 2011). As will be discussed later, with respect to microglia, lacunarity has been associated with changes in the soma and additional morphological features.

Multifractality on the other hand is relevant to patterns in which a spectrum of fractal dimensions can be identified rather than a single global D_B_. The theory and calculations behind multifractal measures are available elsewhere (Chhabra and Jensen, 1989; Smith et al., 1996; Karperien, 2001a).

### Pattern acquisition

For microglia and cells with related morphologies or functions (e.g., neurons, astroglia, dendritic cells, and macrophages), the features usually looked at by fractal analysis are branching patterns and cytoplasmic membrane configuration, but other features relevant to microglia such as nuclear membrane, intracellular structures, textures, movement patterns, or distributions of cells or groups of cells in time and space are amenable to fractal analysis (Losa et al., 1997, 2005; Baatz et al., 2005). In all cases, the feature being investigated has to be converted to a pattern such as a binary image that fractal analysis software can analyze.

#### What you see is what you look for

To convert a chosen structural feature into such a pattern, the feature has to first be revealed by some method that can deliver a signal the software can make sense of. For box counting, as we alluded to earlier, the signal pattern is usually input as a still digital image; that input image is usually made from an original digital image. Microglia are not normally visible, so the digital image is constructed from a histological slide or other visualization method via some amplified signal marker. Such markers are specific for different cell parts and can reveal different features of microglial morphology. Electron microscopy, for instance, reveals exquisite intracellular and the finest membrane detail, the peripheral benzodiazepine receptor, used in live imaging studies, tracks events at the outer mitochondrial membrane, cytoskeletal protein markers reveal differently arranged networks of whichever particular filament is being exposed, antibodies to CD68 show binding on primarily lysosomes in the cytoplasm but also on the outer membrane, RCA-1 shows binding over the entire plasma membrane, and scanning electron microscopy shows gross morphological detail (Streit and Kreutzberg, 1987; Kreutzberg, 1995; Banati et al., 1999; Cross and Woodroofe, 2001). As an example, then, electron microscopy would be more suited to grayscale analysis of intracellular texture and lectin binding to binary analysis of ramification.

Another factor influencing the final pattern of the image is the original size of the region of interest. As their name suggests, *micro*glia are typically smaller than most other cells found in the nervous system (i.e., neurons and other glia). A survey of published values suggests that for human microglia observed at all stages of their activation cycle and the human life span, grown in culture or found in tissue samples, nuclei average roughly 5–10 μm in diameter, cell bodies range from 7 to 20 μm in diameter, and cell branches (if present) radiate outward from the cell body so that an entire cell spans, on average, from 30 to 120 μm (de Groot et al., 1992, 2000; Rezaie et al., 1997, 1999, 2002; Andjelkovic et al., 1998; Sheng et al., 1998a; Stoll and Jander, 1999; Radewicz et al., 2000).

With such small subject matter, it is important to consider the level of detail analyzed and issues such as how well the staining, magnification, and resolution preserved information (e.g., terminal branches or fine membrane fringes). Indeed, despite that fractal dimensions are independent of object size, measured D_B_s are not entirely independent of imaging protocols, including staining methods and image size and resolution, which can affect the results of a fractal analysis to varying degrees. In practice, this limits the applicability of comparing fractal dimensions across paradigms. Over and above absolute differences owing to methodology or image size, however, as is discussed in section “One Fundamental Relative Pattern Across Paradigms,” when looking at branching and membrane detail at least, relative effects are preserved across many paradigms. More work is still needed to characterize issues related to specific methods of visualization and compare how different types of fractal analysis are affected by different visualization techniques.

#### Preprocessing

A major challenge in any type of digital image analysis, pattern extraction itself is another element that must be understood to interpret results of fractal analysis studies. Pattern extraction means removing noise—that can mean overall background and can include other cells or structures. For grayscale box counting analysis, this is often less a challenge than for binary image analysis. Extracting a grayscale pattern is usually relatively straightforward, sometimes involving only isolating the relevant part and converting the original image to grayscale in an image processing program, in which case images of individual cells or entire fields with multiple cells can be processed rapidly. Grayscale analysis assumes that the image background is supposed to be processed along with any objects of interest; if not, grayscale pattern extraction becomes more involved.

Rather than grayscale analysis, however, most of the work published on microglia to date has used binary image analysis. Patterns have been extracted by either manual tracing or automated segmenting methods (Soltys et al., 2001; Jelinek et al., 2008). Both approaches incur their own level of bias. As an illustration, staining in unactivated cells may be splotchier than in activated cells owing to upregulation of the marker on activation, so the operator may interpolate in a “connect the dots” fashion; in automated thresholding, only the actual “dots” are rendered as signal. However, in both cases, a person, either a tracer or a programmer, decides what is background and what is foreground, a universal bias affecting any assessment of staining of biological cells. Also, in automated methods, there is a bias away from overlapping cells so that such methods are usually limited to investigations where clustering and overlap are considered part of the signal rather than noise.

The patterns extracted have typically been profiles or contours of single cells or of fields containing multiple cells. If the goal is to identify branching and membrane characteristics, the patterns generally exclude intracellular content; if the goal is to assess the actual pattern of distribution of a marker, then including inner detail can be appropriate. Depending on the feature being investigated, the final pattern can be filled or outlined; filled patterns of the same cell tend to have higher D_B_s. Ramification has been assessed using another method, skeletonizing (e.g., a function in ImageJ), which is especially suited to the dilation method (Orlowski et al., 2003; Soltys et al., 2005). Of note, the dilation method measures different features of the image so produces different values for the fractal dimension compared to box counting unless the image is Euclidean in which case all fractal dimensions are equal (Jelinek et al., 2005; Losa et al., 2005; Karperien et al., 2008a).

#### Reproducibility

Little has been published on pattern acquisition per se for microglia, but one investigation carried out in our lab compared D_B_s for images of individual microglia obtained using a tracing and a thresholding method (Karperien, 2004). Operator bias within each method was minimizable by having clearly defined rules for the methods, and training operators over a few practice sessions. Although the tracing and thresholding results deviated somewhat from each other for certain images, overall the differences were not statistically significant. That is, the patterns from both methods were not identical, but the results imply that they contained essentially the same information relevant to the D_B_. This investigation was very limited, however, and preprocessing is a contentious issue in digital image analysis (Jelinek and Fernandez, 1998; Jelinek et al., 2005).

Another finding of this work was that D_B_s were statistically significantly lower for images obtained from one compared to many focal planes (Karperien, 2004). This may be attributable to the point that the box counting method used to find the D_B_ from the extracted pattern was intended for 2-dimensional data rather than a sample from 3 dimensions.

Manual tracing methods have the distinct disadvantage of being potentially very time consuming, so have been considered impractical for some types of investigation (Donnelly et al., 2009; Kozlowski and Weimer, 2012). Nonetheless, they are well-suited to investigations of branching patterns and have been used to extract intricate patterns from images of microglia (Soltys et al., 2001; Karperien et al., 2008c).

Semi-automated segmentation methods have also been used (Jelinek et al., 2008; Karperien et al., 2008a); these are quicker, but depend more on suitable starting material. For example, with strong contrast and little overlap of cells an operator can preselect regions of interest, or, alternatively, algorithms have been designed to automatically segment and analyze images of individual microglia or fields of multiple cells without intervention (Karperien, 2001a; Karperien et al., 2008a; Kam et al., 2009). Fully automated segmentation of various shapes is a broad goal in digital image analysis (Kim et al., 2011). Kozlowski and Weimer (2012) have published a promising study in which they demonstrate an automated segmentation method designed specifically for identifying microglia visualized by a variety of protocols, although it is not clear if the method is likely to reveal fine branching patterns; future work is needed to find out if the method extracts patterns suitable for fractal analysis and how generally applicable it is.

#### Validating the result

Another issue one should be conversant in is how well the patterns typically analyzed represent actual microglia. There can be large gaps in the correspondence between overall visual impressions of cells in their original contexts and the final binary patterns, especially when using automated methods geared to identify only pixels corresponding to staining past a certain threshold. Such discrepancies may reflect losses, gains, or distortions of information, or they may be artefacts of perception. That is, regardless of the pattern extraction method used, the essential procedure is to take 3-dimensional, multiply motile, functional cells, and derive from them still, binary, 2-dimensional contours or silhouettes. In addition to containing information about the cell's actual morphology and orientation in space, the information available in the final image will depend on how the original image was acquired and encoded to digital format as well as how the information was extracted. In the case of microglia, detail lost, gained, or distorted could be in intracellular or nuclear features, soma shape, fine branches, membrane protrusions, information about how a cell cuts out its unique volume within the CNS at the moment the image is taken, etc.

Whereas changes to the information may cause the final pattern to bear little resemblance to the original cell or image, this perceptual disjunction has generally been deemed to be negligible based on conventions that have been widely applied to other biological cells. One is the assumption that the information of interest (e.g., in the branching of processes and the contour of the membrane) is sufficiently preserved at least on average (which means sample size and random selection should be considered in any investigation). Another is the assumption that objects contained in 3D without completely filling 3-dimensional space are adequately represented by projections onto 2-dimensional space (e.g., theoretically, a contour of a tree's branches should hold the same information as the tree) (Takayasu, 1990; Vicsek, 1992; Smith et al., 1996; Losa et al., 1997; Jelinek and Fernandez, 1998; Fernandez and Jelinek, 2001). As discussed in the next section, computer based modeling has been used to test the validity of these assumptions for branching patterns in microglia.

#### Microglia *in silico*

Modeling lets us test our understanding of and predict the behavior of biological systems. Work by Jelinek et al. (2002) and others from that laboratory demonstrated that increasing ramification in microglia can be modeled by increasing complexity. Changes in complexity input to models can, in turn, be accurately measured back from binary outlines extracted from *in silico* microglia. Jelinek's team created computer-simulated models from images of microglia in normal and pathological elderly human brain, as well as several other samples from humans and other animals. The models were specified by recursively applied rules derived from measuring microglial features. These included features associated with fractal geometry, such as process length and diameter, branching frequency, and branching angle, as well as other features, including the tortuousness of processes and size and shape of the soma, that were expected to influence the results. The models were generated as several 200-member populations of both idealized models and statistical models designed to emulate microglia in the 3D space they occupy under real circumstances, or at least in observed circumstances (Karperien, 2001c, 2004; Jelinek and Karperien, 2008).

**Figure 5:**
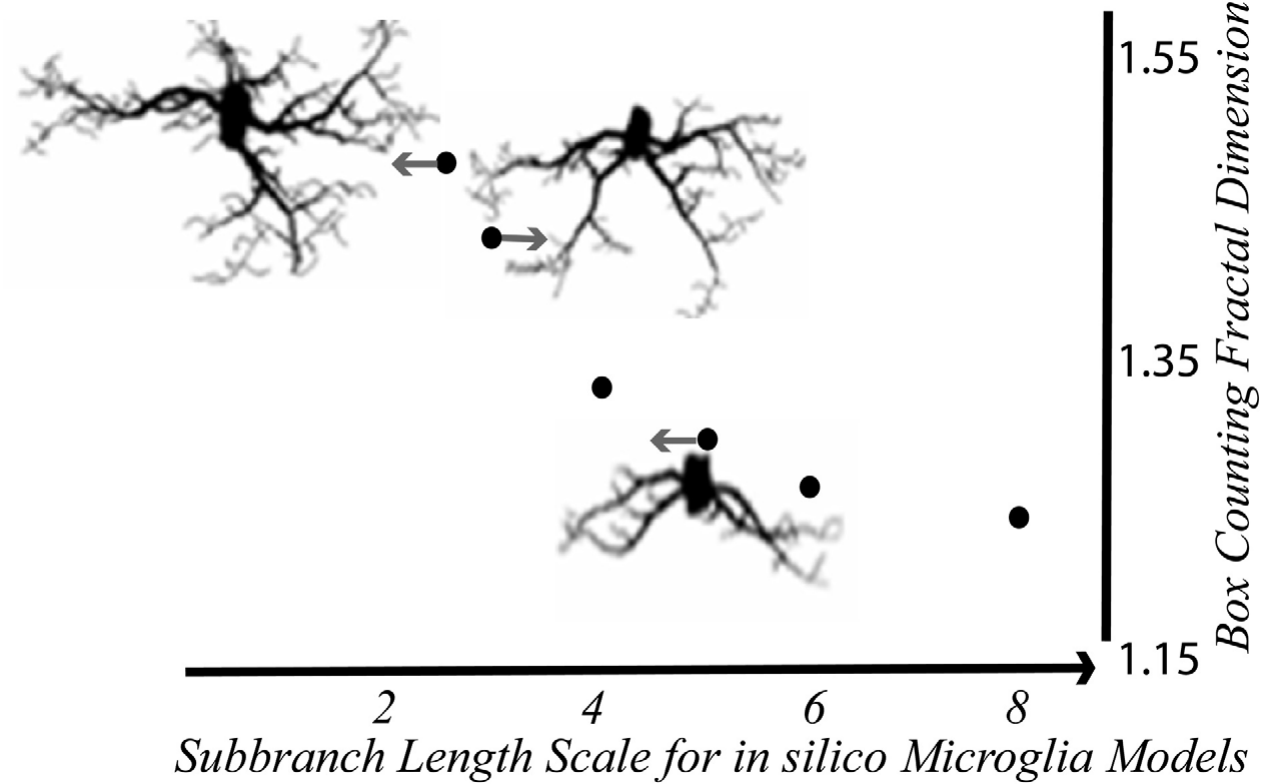
***In silico* microglia illustrate the relationship between the D_B_ and branching features**. The D_B_ decreased with scale—that is, it increased nonlinearly with the relative length of sub-branches Modified from Karperien (2004); models generated with MicroMod (Karperien, 2001c; Jelinek et al., 2002).

Supporting the conclusion that binary contours from microglia realistically represent underlying scaling features, the average D_B_s of binary contours extracted from the model populations corresponded to the D_B_s that would be expected for theoretical fractal microglia having the same underlying scaling features (see Figure 5). Ramified microglia modeled on a population with a mean D_B_ of 1.423 for instance, had a mean D_B_ of 1.425. Furthermore, the D_B_s changed as would be expected for fractal patterns when the length or diameter of the modeled branches changed, but were not affected when the soma size or elongation changed. The results were robust under the emulations of 3D space, but only within limits (Jelinek et al., 2002; Karperien, 2004; Karperien et al., 2005; Jelinek and Karperien, 2008).

Overall, the *in silico* studies suggest that, in practice, some variation in calculated D_B_s is predictable even for cells in equivalent activation states having essentially the same branching ratios, attributable to the space the microglia occupy, and the orientation they assume at any point in time. The variation not related to scaling is small enough to conclude that binary contours represent microglial morphology for box counting fractal analysis, at least for the particular pattern extraction methods and large sample sizes used. However, a feature of the models that limits the applicability of these results is that the modeller could control the background and the degree of “staining,” and the models had very high contrast (Jelinek et al., 2002).

## What fractal analysis has told us

The preceding section outlined what fractal analysis is and provided some fundamental information to help the reader understand how box counting analysis is applied and gain insight into interpreting it when applied to microglia in particular. The present section outlines some of the key results that have been obtained for investigations of branching pattern and membrane detail, and discusses issues in understanding how they all fit together and directions they suggest for the future.

### One fundamental relative pattern across paradigms

Mostly in agreement with modeling results and fractal theory, the results of fractal analysis studies using box counting and other techniques support the broad idea that the D_F_ for overall microglial morphology increases with ramification (but, as discussed below, does not necessarily change inversely with activation state) (Soltys et al., 2001, 2005; Orlowski et al., 2003; Jelinek et al., 2008; Karperien et al., 2008c). This was seen for a variety of image sizes, resolutions, staining methods, species, and brain locations. One study in particular compared several paradigms, and found that despite there being differences in the actual values obtained for the D_B_, a basic relative relationship was preserved over many protocols, suggesting there is a fundamental pattern transcending many factors (Karperien, 2004).

One investigation (Jelinek et al., 2008) using cultured rodent cells demonstrated the general pattern with the D_B_ corresponding to responses to graded levels of a stimulus with de-ramifying on one side of a cycle and re-ramifying on the other. The general pattern emerges in graphics when cells with the lowest D_B_, approaching theoretical values for simple shapes like circles (i.e., 1.0), are placed at the bottom, and cells with the highest D_B_ (i.e., the most complex) are placed at the top. This basic pattern was mapped in Figure 1. The figure is illustrative rather than strictly accurate in that it uses only a few representative morphologies arranged by their corresponding D_B_s but not necessarily separated by re- vs. de-ramifying or pathological vs. normal status.

#### The cycle of complexity is not quite as expected

Empirical results add to the fundamental relative pattern that “most activated” means “least ramified,” but “least activated” is not necessarily “most ramified.” This is because the most complex microglial form seems to be ramified cells that are subtly activated.

Thus, the evidence suggests that the basic pattern is characterized by an initial increase in the D_B_ when unstimulated ramified cells start responding, followed by a decrease until the roundest forms are reached, then an increase back up to the ramified states. The amount of data available at this point is insufficient to establish normative ranges, but, as a rough guide and taking note that the actual values varied with the investigative protocol, the peak in D_B_ was typically around 1.42, and rarely above 1.50. The essential cycles illustrated in Figures 1 and 7 thus can be tentatively understood as cycles going from 1.00 at the bottom to 1.50 at the top, but establishing normative ranges for ramified cells is another challenge for future fractal analysts (Soltys et al., 2001, 2005; Karperien, 2004; Jelinek et al., 2008; Hinwood et al., 2012). Various studies have shown that the cells with the peak values for complexity are both highly ramified and in compromised environments (i.e., ramified cells in uncompromised environments had slightly lower D_B_s). In some studies these were cells with many medium to long, hypertrophied processes. This configuration may be in addition to, or related to, a phenomenon known as “hyper-ramification,” due to ramified cells subtly responding to noxious stimuli such as chronic stress (Hinwood et al., 2012).

Potentially relevant to quantitating hyper-ramification is that many methods depend on changes in markers constitutively expressed at low or undetected levels then upregulated during responses to compromise such as chronic stress. This opens up the possibility of morphological information being underrepresented in ramified cells being used as comparisons to identify activated cells. Further research about the extent to which a cell has become hyper-ramified, vice having possibly had more existing detail made visible (e.g., as revealed by upregulated MHC-II in the absence of actual morphological changes) is required (Kanaan et al., 2010; Kettenmann et al., 2011; Hinwood et al., 2012).

#### Differences according to experimental paradigm

Over and above the finding of a fundamental relative pattern hangs the issue of the actual differences in fractal dimensions found with different investigations. There are important practical considerations in fractal analysis to consider that may underlie such differences. Accordingly, below we discuss several factors affecting microglial morphology that may be relevant to fractal analysis.

**Figure 6:**
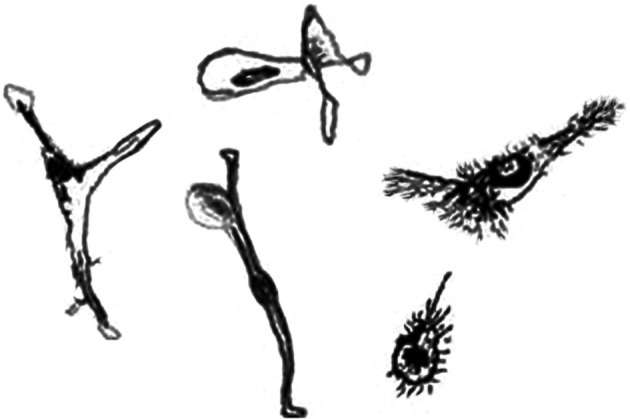
**Drawings of representative microglia grown in the laboratory**. Cells grown in culture tend to appear different than microglia found in live animals. Art by Thomas R. Roy.

***Downward shift in D_B_ for microglia grown in the laboratory.*** The D_B_ for cultured microglia showed the fundamental relative pattern for microglia described above, but actual values tended to be lower than for other protocols. This may be related to certain differences that distinguish these cells from other preparations due to the culture environment and have the potential to affect the results of fractal analysis. Such cells, depicted in the drawings in Figure 6, typically look different from other microglia. On ramified cells, secondary branches are often less apparent or appear as extremely fine extensions, and cellular contours generally appear smoother. Lamellopodia are frequently more evident, and hairy-looking fringes, not normally detected in light microscope examinations of microglia from tissue, may be seen. The drawings show morphologies from unstimulated (left) to becoming activated (right) but lack an initially rounded morphology that immature cells in culture often have before ramifying. In addition, depending on many conditions, cultured microglia adopt other morphologies not shown in these drawings (de Groot et al., 1992; Bohatschek et al., 2001; Kettenmann et al., 2011; Olah et al., 2011).

Some of the differences such as seen with lamellopodia translate into less detail than is typical of microglia in tissue and lead to a lower D_F_ for ramified cells in particular. Our lab did find in one investigation that the D_B_ of cultured cells increased as subjective ratings of activation increased. This result was attributed to a technical issue that rendered secondary branches invisible on many of the ramified cells (Karperien, 2004). The cytoskeletal reorganization seen in microglia is known to distinguish microglial ramification from ramification of other cells, so it might be informative to separate cytoskeletal rearrangements from strictly membrane features (Faff et al., 1996; Tanaka et al., 1999; Faff and Nolte, 2000; Ohsawa et al., 2000). Perhaps comparing cytoskeletal components in cultured and *in vivo* microglia would eliminate the difference, or perhaps the difference is indeed inherent to the spatial context of microglia in the culture environment. In this respect, there are a host of factors that influence microglial morphology in culture (e.g., the presence of astroglia). In general, work is needed to confirm if the D_F_ is lower in other types of fractal analysis and to further clarify quantifiable differences between *in vitro* and *in vivo* morphologies with respect to different microglia classes and interpretation of microglia cycling.

***The unknown influence of species on the D_F_.*** Another element that may have been a factor in different studies reporting different values from fractal analysis is species. Microglia are presumed to reside in all mammals and have been found in several other vertebrates and invertebrates (e.g., birds, frogs, fish, snails, and leeches) (Dowding et al., 1991; Dowding and Scholes, 1993; Sonetti et al., 1994; Magazine et al., 1996; Dobrenis, 1998; Bernhardi and Nicholls, 1999; McMenamin, 1999; Navascues et al., 2000), which has led many researchers to generally classify microglial morphology assuming the fundamentals apply across species. But this assumption is questionable because microglia from one species are not necessarily comparable to microglia from another. In some cases, significant differences in various features have been noted even between strains of one species (Humphrey and Moore, 1995; Klyushnenkova and Vanguri, 1997). Microglia differ in how they are distributed in any particular species (Hutchins et al., 1990; Andjelkovic et al., 1998; Maslinska et al., 1998; Rezaie and Male, 1999; Rezaie et al., 1999; Navascues et al., 2000; Male and Rezaie, 2001); the evidence suggests that some amphibians and rodents have smaller, and some fish considerably larger, proportions than humans (Lawson et al., 1990; Dowding et al., 1991; Sonetti et al., 1994). Other differences have been found in function and staining, as well as fundamental morphology (Chen et al., 2002; Hayakawa et al., 2005; Jinno and Yamada, 2011). A difference affecting ramified microglia in particular is that processes branch subtly differently according to species (Finch et al., 2002). Broadly speaking, any of a host of species-related differences in microglia may have accounted for some of the difference across paradigms, but the significance of species differences for fractal analysis is unknown. A future challenge for fractal analysts, then, is to explore differences amongst microglia from different species.

***Branching angle.*** The difference noted above in microglial branching angle with species deserves further consideration. It is known that microglia tend to sprout essentially orthogonally near their middle and end, filling the outer portion of the spread of processes with nearly perpendicular branch points. They are more orthogonal than astroglia, for instance, which tend to branch more acutely (Karperien, 2001c, 2004; Jelinek and Karperien, 2008). Branching angle in general may have special relevance to fractal analysis of microglia, because not just the degree of branching but in particular branching angle influences both flow within branched structures and the D_B_ (Hahn et al., 2005).

***Potential influences of texture and space.*** Another factor that may have contributed to differences in the D_F_ is texture. Microglia respond to textures such as glass and various biomaterials (Dobrenis, 1998; Tanaka et al., 1999; de Groot et al., 2000; Wollmer et al., 2001). Research into mechanisms behind the rejection of biomedical implants has shown that microglia respond to coatings used on electrodes and implants, for instance (Leung et al., 2008). Perhaps future applications of fractal analysis will move our understanding of biomedical implant rejection forward, but whether or not fractal analysis is sensitive to the effects of various textures is currently unknown.

Space may also have accounted for some differences. Some morphological variability of microglia grown in the laboratory and identified in tissue is known to be accounted for by 3-dimensional space and how microglia orient themselves in it. Given room, for instance, the processes of microglia grown in culture tend to avoid each other, and under astrocyte layers, whole microglia appear flatter than cells above, and rounder in more open than confined spaces (Dobrenis, 1998; Tanaka et al., 1999; de Groot et al., 2000; Wollmer et al., 2001). A topic currently being investigated is how microglia in the human CNS are oriented with respect to each other and with respect to neurons, and how this influences brain function (e.g., in autism in humans). Microglia are more dynamic in space and time than neurons and may therefore play important roles in real-time signal processing (Morgan et al., 2012). One study has used both 2D and 3D data to analyze the D_F_ of microglia in pathological scenarios, and as this technology continues to evolve (Sheets et al., 2010; Xiao et al., 2011), fractal analysis may prove valuable for modeling and understanding the significance of orientation in space and interdigitation amongst these mutually-existing branched structures (Pow et al., 1989; Hahn et al., 2005). Currently, however, this is another topic that needs to be explored.

***Location and subtype.*** Other factors that affect microglial morphology are location and subtype. In CNS tissue samples, microglia adopt different conformations depending on where they are from and local characteristics within that area, to the extent that investigators formally classify subcategories based on location and different staining characteristics (Lawson et al., 1990; Mittelbronn et al., 2001). For instance, whereas microglia in gray matter are usually more stellate, hugging neuron cell bodies, sprawling alongside of oligodendroglia, or intertwining with astroglial processes, when in intra- and inter-cortical tracts, they are more often found in a bipolar, lengthwise orientation with a leading process, suggesting they may be in motion and giving them the appearance of a less ramified cell even though they are not “activated” (Kreutzberg, 1995; Rezaie et al., 1997; Rezaie and Male, 1997, 1999; Bayer et al., 1999; Radewicz et al., 2000; Male and Rezaie, 2001). Perivascular microglia, too, adopt conformations distinguishing them from other microglia. Characteristically elongated and conforming to the surfaces of the vessels they lie along like lichens clinging along branches, they are less stellate than ramified microglia networked throughout the CNS. A relatively sprawling and untortuous morphology of ramified microglia can be observed in the retina, where they may be found in a horizontal laminar distribution from the nerve fiber layer to the outer plexiform layer (Liu et al., 2012). One pilot investigation of differences reported in the literature suggests the D_B_ can be affected by the location at which microglia are found in the brain (Karperien, 2004). More and more phenoytpes of microglia are being proposed (Olah et al., 2011) and the details of such differences still need to be charted.

### The language of morphological classification and how it relates to the D_B_

**Figure 7:**
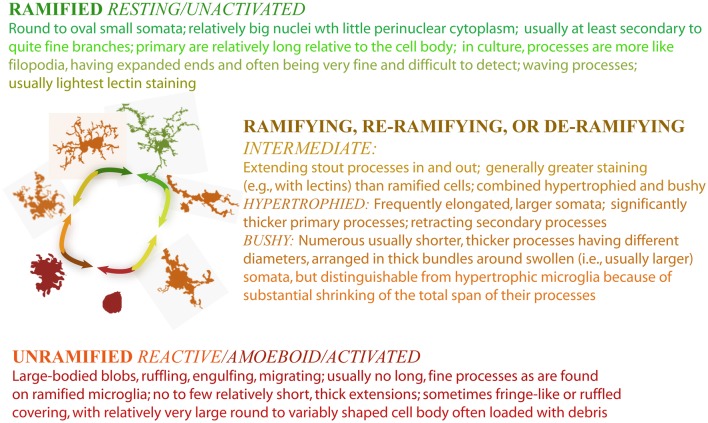
**Criteria for morphological categories of microglia**.

In addition to characterizing how the D_F_ changes with morphology, research has looked at how the D_F_ corresponds to subjective morphological categories. Underpinning virtually all recent microglial research is the system of morphological/functional categories we discussed in the introduction to this review. In the scientific literature, people describing microglial morphology use primarily words grounded in this model of *reactive microgliosis*, a term for the response of microglia to compromise in the CNS (Streit, 2000). Typically, this system includes ramified and activated categories and forms between, which have variously been referred to as “intermediately activated,” “bipolar,” “rod-like,” “hypertrophied,” and “bushy.” (Note that “rod” cells are presumed to be fused microglia) (Ziaja and Janeczko, 1999; Streit, 2000). Along these same lines, Figure 7 lists an operational definition used in research carried out in our lab (Karperien, 2004). The list synthesizes descriptions from various authors of what microglia look like at places along the cycle. Investigators commonly use systems of two, three, or four categories according to their needs, and usually specify features of each category in their operational definitions but do not always publish their precise definitions.

With no classification standard, from one published study to the next, definitions of microglial morphology differ, categories overlap, and comparisons are awkward. Moreover for people applying objective criteria in order to classify microglia, shifts and overlaps in categories are often evident but difficult to quantify. At worst, microglia may be assessed using techniques and categories insensitive to certain subtle variations and disruptions that may have grave consequences for people harboring them. Thus, quantitating that model is of great interest in neuroscience.

#### D_B_ discriminates a 4-category classification

Studies of how D_F_s correspond to visually assigned morphological categories have so far yielded three main results. (1) Fractal dimensions discriminated between all categories in four-category systems. (2) D_F_s did not consistently discriminate three-category systems, failing to differentiate intermediate from ramified cells in some studies. And (3), despite failing to discriminate three-category systems, they nevertheless discriminated other subtle and visually undetectable differences within a category, ramified normal and ramified compromised (Soltys et al., 2001; Karperien, 2004; Karperien et al., 2005).

These results suggest that classifying microglia using a three-category system ignores differences in complexity that a four-category system detects. That is, where people subjectively group some “bushy” and some “hypertrophied” cells with ramified, the D_F_ objectively draws lines quantitating these categories (as well as activated). Moreover, that fractal dimensions discriminate between subtle differences within ramified cells from compromised vs. normal tissue suggests a fifth category, presumably the peak cells noted above, may be quantifiable.

These results may be particularly relevant to counting cells classified into a category. Because the changes in microglial morphology occur in so many domains and reflect so many influences and events, finding the same number of cells in a morphological category might obscure subtle but important differences between samples in what the cells are doing. By clarifying the functional and morphological correlates of differences in complexity within categories, fractal analysis may be a major contributor to making models of microglial function more quantitatively sensitive to subtle but meaningful changes in morphology. Further study of the quantifiable differences may also lead to identification of rules that will assist human observers in categorizing microglia.

### Quantitating responses

Another potential application of fractal analysis is objectively quantitating effects of treatments without classifying cells. Researchers have successfully used D_F_s to quantitate responses of cultured microglia to different treatments including the addition and removal of as well as graded levels of activating stimuli (Jelinek et al., 2008), subtle effects of LPS and naloxone when those effects were not visually detectable (Karperien et al., 2008a), and nonsteroidal anti-inflammatories in models of age-related disease (Varvel et al., 2009).

Other investigations have demonstrated the potential to use fractal analysis for quantitating pathological status based on the average D_F_ and on the overall distribution of complexity in samples containing microglia (Soltys et al., 2005). One pilot study (Karperien, 2004) indicated that the D_B_ clustered within distinct ranges respectively for control, acute, and chronic responses to spinal cord injury. This was based on a very small convenience sample, so the results should be considered with caution. Another study showed that for microglia in postmortem tissue, the average D_B_ distinguished control from both overtly and subtly pathological human tissue (Karperien et al., 2008c). Additional work suggests that the distribution itself of complexity can provide important clues about pathological status, where rather than an average, the proportions of cells with certain constellations of quantitative features may provide information about what is happening in a particular location at a particular time (Karperien, 2004).

#### Injury, disease, and drugs

Further work is necessary to verify and expand this small body of work, but the results so far suggest that the discriminating capacities of the D_B_ may be powerful for gauging microglial activation in incipient or ongoing responses associated with injury, disease, and drug use (Soltys et al., 2005). This could affect clinical decision-making as well as laboratory work. Based on the work described above, one application that could be explored is measuring the fractal dimension of microglia in spinal fluid to monitor and quantify pathological status, stages of an ongoing response after injury, or effects of drugs in patients (e.g., for schizophrenia) (Nikkila et al., 1999; Stoll et al., 2006; Blackbeard et al., 2007). Another area we can speculate that fractal analysis of microglia may be helpful in is in making mathematical models to predict drug responses. Whether or not average D_B_s or profiles of the distribution of complexity will be useful for differentiating different diseases or states by quantifiably characterizing the numbers of various types of morphology without the need for subjective classification is a question for future research.

#### Tracking microglial morphology

Another area with promise for the future is building on recent work in tracking microglia with *in vivo* imaging.

***Diabetes.*** In diabetes and glycemic status in human and animal models microglia have been implicated as playing important roles in pathological sequelae (Chahed et al., 2010; Polito et al., 2011). Liu et al. (2012), for example, have tracked the association of retinal microgliosis with retinal ganglion cell degeneration in rodents. Several studies of rodent models of diabetes have shown that the function and morphology of microglia in different areas of the CNS can be affected (e.g., marked shortening of processes) by compromised glycemic status without cells becoming activated (Gaucher et al., 2007; Arden and Sivaprasad, 2011). Based on work cited earlier in this paper describing how similar changes affect the D_B_, it is likely that the types of changes that have been noted in diabetic sequelae are likely to influence the D_B_, suggesting another potential use of fractal analysis that could be developed. This may be especially relevant with respect to diabetes research for live monitoring, but will depend on *in vivo* visualization techniques being further developed.

***Alcohol and thiamine deficiency.*** The link to diabetes research brings up the point that diet and nutritional status in general are factors that can have multiple, compound effects on microglia. It is well documented that a host of factors in the milieu affect microglia (e.g., acidity and temperature) (Faff and Nolte, 2000; Rezaie et al., 2002). Researchers have shown that microglia in mice respond rapidly to a high fat diet by infiltrating the hypothalamic arcuate nucleus (Yi et al., 2012). Effects on microglia of thiamine deficiency, which may occur in alcoholics or nutritionally deprived people, have also been studied. Like all cells, microglia depend on energy, thus can become dysregulated by metabolic compromise (e.g., ATP may be unavailable or lactate may accumulate with reduced thiamine dependent enzymes) (Park et al., 1999). Researchers have shown that microglia in rats are exquisitely sensitive to such compromise, changing both their profile of proteins expressed and their morphology (Zhao et al., 1996), but not necessarily being classifiable as responding. It has been reported, for instance, that with thiamine deficiency, especially perivascular microglia become “plump,” although still have processes, prior to and probably mediating eventual overt neuronal damage (Sonetti et al., 1994; Dickson, 1999; Todd and Butterworth, 1999a,b; Calingasan and Gibson, 2000a,b). Again, because the changes noted are subtle and difficult to objectively classify but also the kinds typically quantifiable by the D_B_, this may be another area that could be explored by fractal analysis.

### Age-related changes

#### Microglial morphology in the very young: from rounded to ramified

Another subject in which fractal analysis has been used to quantitate typical changes in microglia is early development. Microglia play many roles in neurodevelopment, at different times, places, and forms. Proposed roles include “fine-tuning” CNS structure, promoting axonal growth, directing neuronal migration, determining neuronal phenotypes, interacting with oligodendrocytes in myelination, influencing vascularization, and disposing of debris and normally dying cells. Through epigenetic, structural, and other mechanisms, they may also contribute to developmental disruptions and subsequent neuropathology such as in fetal alcohol spectrum disorder, cerebral palsy, Down's syndrome, autism, and schizophrenia (Hao et al., 2001a,b; Aarum et al., 2003; McAllister and Miller, 2010; Maezawa et al., 2011; Paolicelli et al., 2011). There are many opportunities to test the ability of fractal analysis to contribute to our knowledge of early development.

In humans, rather than there being one general early microglial morphology, there are characteristic temporal and spatial patterns. Many of these appear to be very much the purview of fractal analysis. For instance, microglia appear very early and then colonize the CNS mainly during the second and to some extent the third trimester of fetal development (in rodents, microglia start to populate the CNS around or after birth). In tissue from humans gestated a few weeks, investigators first see “rounded” cells, then “intermediately ramified” cells migrating along white matter tracts and blood vessels; then, later in development, mainly fully ramified cells. This basic temporal pattern is superimposed on several spatial patterns—different brain areas are populated at different times but according to usually the same basic morphologic sequence. Orlowski et al. (2003) have demonstrated an elegant quantification of this progression using fractal analysis as cells becoming increasingly complex over time. There is evidence that distinct morphological and functional subtypes of microglia appear even in the earliest stages of development, which requires further investigation (Hutchins et al., 1990; Andjelkovic et al., 1998; Cuadros and Navascues, 1998; Maslinska et al., 1998; Alliot et al., 1999; Navascues et al., 2000; Rezaie et al., 2002).

Another topic that could be explored with fractal analysis is variation in different areas. Billiards et al. (2006) have found that human fetus and infant cerebral white matter is densely populated with “intermediate” and “amoeboid” microglia and that there is a transiently increased density of activated microglia (i.e., cells in the typical morphology marked by CD68) in the cerebral white matter prior to 37 weeks gestation. This peak has been proposed to translate into a window of increased vulnerability to injury mediated by microglia (e.g., hypoxic injury underlying cerebral palsy). Fractal analysis may add to our knowledge by quantitating such differences and providing a finer level of knowledge about what cells are doing in different places and times in development.

Rounded cells seen early on in development are generally considered to be “immature” rather than activated. Many authors call a cell “amoeboid,” regardless of its origins or maturity, as long as its cell body is amorphous, long, fine processes are absent, and pseudopodia are (usually) present, but other authors object, saying that immature and unbranched as opposed to reactive microglia differ. It is not clear if the microglia that are first seen as rounded cells are rounded because they are immature or because that is the morphology in which they are best able to migrate through, prune, and clean up the developing CNS. Further, the extent to which microglia deramify and reramify in developing CNS is not known. These are questions for which fractal analysis may be able to provide additional insight.

#### Microglial morphology in the very old

Not only do microglia change over early development, microglia in the very young are not the same as microglia in the very old. Microglia generally increase from sparse cells with few branches in infancy to a ramified network in adulthood to an increasingly dense network of increasingly “reactive looking” microglia in old age. Primary microglial processes are usually shorter, thicker, and more numerous in adults as compared to infants, and again in nonelderly adults compared to elderly adults. Researchers have also characterized a tendency toward more aberrant features, including twisting and fragmenting of processes, with age (Sheng et al., 1997a,b, 1998a,b; Rozovsky et al., 1998; Sheffield and Berman, 1998; Nichols, 1999; Sheffield et al., 2000; Finch et al., 2002; Yu et al., 2002; Olah et al., 2011).

Within the overall spectrum of age-related changes, other factors such as gender and brain region can further influence microglia and may be reflected in the morphology itself or at least how that morphology is revealed under the microscope. To illustrate, some researchers have found that HLA-DR expression is higher in normal elderly human males than females (but higher in females with Alzheimer's disease), and significantly so only in white matter. In a study of rodents, Morgan and colleagues (1999) found that food restriction lessened age-related increases in complement receptor expression but not MHC-II expression in microglia in the basal ganglia, but food restriction and age did not affect these two measures in the outer molecular layer of the dentate gyrus (Kreutzberg, 1995; Carson et al., 1998; Hurley et al., 1999; Morgan et al., 1999; Overmyer et al., 1999; Ren et al., 1999; Wierzba-Bobrowicz et al., 2000a,b; Cross and Woodroofe, 2001; Kanaan et al., 2010).

The changes of age are accompanied by functional changes in elderly adults, whereby from development to old age, microglia lose their ability to protect and become more likely to react or more likely to react abnormally. Why microglia become “senescent” or “dystrophic” is unknown, but it has been suggested that the changes may be at least partly mediated by and contribute to the changing hormonal environment of aging as well as a reflection of accumulating pathology.

Modeling studies suggest that the changes described in the literature would affect the D_B_. For instance, in space-emulating models simulating process swelling in isolation from other changes—i.e., changing only the diameter of primary branches—as process diameter increased relative to soma diameter, the measured D_B_ decreased despite the fact that the complexity input to the models did not. Another factor relevant to age-related changes that affected results was the number of primary branches. The D_B_ tended to increase with the number of primary branches even when the fundamental complexity of the branches was not changed. Tortuousness was another factor having an effect: in keeping with results for known fractal patterns, the average D_B_ for more tortuous models generally deviated more from theoretical than for straighter patterns (e.g., ~5 vs. 3%) and models with curled rather than sprawled processes had higher D_B_s (Jelinek et al., 2002; Karperien, 2004; Karperien et al., 2005; Jelinek and Karperien, 2008).

Little research has been published describing actual measurements of these changes with fractal analysis. One investigation measuring the D_B_ for microglia from elderly human brain illustrated that the distribution of complexity in healthy elderly human brain differed from that for pathological elderly human brain (e.g., for the D_B_ range from 1.30 to 1.43, pathological brain had more cells, and for the range from 1.40 to 1.48 healthy elderly had more), and that microglia in the healthy brain had a different complexity profile than more typically noted for younger adult brain (Karperien, 2004). Future research exploring age-related changes in microglia and correlates in the D_B_ may shed light on the topic of age-related disease and dysfunction.

### Results of supplementary measures

#### Lacunarity and other morphometrics

Virtually all of the fractal analysis studies in the literature compared D_F_s against other metrics. The utility of the D_F_ varied with different methods. In many cases where multiple measures were compared, the D_F_ was the most consistently useful measure of, and in some cases, the only measure refined enough to detect, the most subtle and difficult to identify changes. One consistent result was that D_F_s were generally complemented by various measures of lacunarity, where using them together gave the most sensitive correlation to morphological categories (Soltys et al., 2001, 2005; Orlowski et al., 2003; Karperien, 2004).

In some work, measures of the size and shape of the 2-dimensional space occupied by a cell were less sensitive to differences in microglial morphology, where the D_B_ was superior to Λ in detecting the subtlest changes, but in some studies Λ was shown to be especially good at detecting changes in soma shape and size that D_F_s did not detect (Soltys et al., 2001, 2005; Orlowski et al., 2003; Karperien, 2004).

Some investigations found some D_F_s to be correlated with certain features, but the D_B_ in particular was generally deemed not superfluous with other measures. In one study, the D_B_ was correlated with Λ, but only for cells at certain levels of activation. The DB was correlated with the number of pixels in some cases, but generally not strongly correlated with the density of pixels, and the number of pixels was not as useful an index of function-related change as was the DB. Modeling studies showed that Λ was sensitive to many of the changes affecting the D_B_ but as an independent measure; the two were not consistently correlated, except for tortuousness, for which they were strongly negatively correlated (Karperien, 2004; Karperien et al., 2008c; Jelinek et al., 2011).

#### Multifractality and local dimensions

Modeling and other studies using multifractal measures suggest mutlifractality and local dimension analysis may be useful for identifying particularly intermediate forms of microglia. Modeling studies have shown that microglia modeled with the same branching parameters on all branches did not scale as multifractals, but models with disparate parts were more likely to scale as multifractals and showed variation in the local dimension. These results are consistent with other results (Karperien, 2004; Karperien et al., 2011; Jelinek et al., 2013) indicating that multifractal scaling is rare overall but that ramified microglia are more likely than others to show multifractal scaling, and when they do, to have disparate parts. One challenge for the future, then, is to clarify the nature of multifractal scaling in microglia, and pursue the potential for multifractal analysis to reveal cells in a transitional state having elements of more than one typical level of activation.

## Summary and conclusion

In summary, microglia have emerged as tiny wielders of formidable power and are now serious targets for study and intervention in essentially anything that goes on in the nervous system. We know well that their form and function are tightly coupled, but to take our investigations and therapies to the next level, we need to know this more quantitatively. An obstacle to moving forward is the very nature of microglial morphology. Microglia undergo changes in a host of domains along multiple time scales, changes that are indeed challenging to distil into practical metrics. Fractal analysis, a well-established technique in neuroscience, is coming into its own as a tool to help quantitate our models of microglial form and function. Able to quantify not only gross morphological differences but also subtleties of microglial morphology that may hold clues to understanding normal and pathological CNS, it has provided a fundamental quantitative model relating complexity to morphology and has provided insight into how to quantitate and improve our classification systems.

It has begun to provide insight into how to answer the questions put to the reader at the beginning of this review, and in the future, combined with measures of other features from complementary morphological analysis techniques may provide full answers. Questions posed about the point at which to consider a cell to have crossed over from ramified to activated, or about whether it is de- or re-ramifying, for instance, might be investigated by combining live video techniques with the power of fractal analysis to discriminate and quantitate subtle morphological differences. Questions posed about subtle differences in branching angle and other features not accounted for in traditional classification systems might be investigated through fractal and multifractal methods combined with traditional metrics such as soma size. Questions about interpreting the distribution of different morphologies or other features of microglia in space might also be investigated using fractal analysis of sections and grayscale analysis rather than individual cells in binary images.

As noted throughout this paper, the future holds many challenges on several levels. Theoretically, 3-dimensional and 2-dimensional methods should be comparable, but this remains to be tested. Key issues that need to be explored in the laboratory are the application of 2-dimensional fractal analysis methods to live video monitoring and comparisons to 3-dimensional fractal analysis methods in order to quantitate and understand microglial form and function in real time and space. Nonetheless, complexity measured by box-counting fractal analysis of 2-dimensional images has been shown to be a robust and powerful measure of the subtlest changes in one feature of microglial morphology that is associated with function. Basic groundwork has been laid to move forward in some areas and explore the utility of fractal analysis in clinical applications where these subtlest of changes may matter such as tracking disease progression, healing after trauma, drug responses, implant rejection, etc. Indeed it may be the case that clinical application will precede full knowledge of the implications of fractal analysis.

### Conflict of interest statement

The authors declare that the research was conducted in the absence of any commercial or financial relationships that could be construed as a potential conflict of interest.
